# Bromodeoxyuridine does not contribute to sister chromatid exchange events in normal or Bloom syndrome cells

**DOI:** 10.1093/nar/gkw422

**Published:** 2016-05-16

**Authors:** Niek van Wietmarschen, Peter M. Lansdorp

**Affiliations:** 1 European Research Institute for the Biology of Ageing, University Medical Center Groningen, University of Groningen, 9713 AV Groningen, The Netherlands; 2 Terry Fox Laboratory, BC Cancer Agency, Vancouver, BC V5Z 1L3, Canada; 3 Division of Hematology, Department of Medicine, University of British Columbia, Vancouver, BC V6T 1Z4, Canada

## Abstract

Sister chromatid exchanges (SCEs) are considered sensitive indicators of genome instability. Detection of SCEs typically requires cells to incorporate bromodeoxyuridine (BrdU) during two rounds of DNA synthesis. Previous studies have suggested that SCEs are induced by DNA replication over BrdU-substituted DNA and that BrdU incorporation alone could be responsible for the high number of SCE events observed in cells from patients with Bloom syndrome (BS), a rare genetic disorder characterized by marked genome instability and high SCE frequency. Here we show using Strand-seq, a single cell DNA template strand sequencing technique, that the presence of variable BrdU concentrations in the cell culture medium and in DNA template strands has no effect on SCE frequency in either normal or BS cells. We conclude that BrdU does not induce SCEs and that SCEs detected in either normal or BS cells reflect DNA repair events that occur spontaneously.

## INTRODUCTION

Sister chromatid exchanges (SCEs) are a possible outcome of DNA double-strand breaks (DSBs) that were repaired through homologous recombination. As such, SCEs are considered sensitive indicators for genome instability in cells (1). This is evidenced by increased SCE rates observed in cells treated with mitomycin C (2), exposed to X-rays (3) or ionizing radiation (4). SCEs are classically detected cytogenetically by means of differential staining of sister chromatids in metaphase spreads from cells cultured with BrdU for two cell cycles. Visualization of chromosomes by staining with Hoechst or Giemsa allows for differentiation between sister chromatids for which either one or both DNA strands are labeled with BrdU (2,5,6). However, it has been widely reported that SCEs are induced by culturing cells in the presence of BrdU, raising questions about if and how many SCEs occur naturally during the cell cycle (7–11). This holds especially true for cells derived from Bloom syndrome (BS) patients.

BS is a rare recessive genetic disorder caused by mutations in the *BLM* gene (12). This gene encodes for the BLM protein, which is a member of the RecQ family of helicases and plays an important role in preventing SCE formation during repair of DSBs (13). Cells from BS patients display marked genome instability, evidenced by the high SCE rates (12), as well as delayed speed of DNA replication and cell division (14), elevated mutation rates (15) and disrupted nuclear architecture (16). In the case of BS, it has been reported that the characteristic high SCE rates only occur during the second DNA replication, when BrdU labeled DNA is used as a template for DNA replication (17,18). Conflicting reports state that the high SCE rates in BS cells do occur spontaneously (19,20).

We recently described a new technique for SCE detection, called Strand-seq (21). This technique relies on single-cell DNA template strand sequencing to detect chromosomal rearrangements, including SCEs. One of the major strengths of Strand-seq is that it only requires one round of cell division in the presence of BrdU, thus eliminating any effect of DNA replication using BrdU-labeled templates. Furthermore, Strand-seq allows mapping of SCEs at kilobase resolution or higher, which is several orders of magnitude better than detection by cytogenetics.

We used Strand-seq to study SCE rates in both normal and BS cells to elucidate the effect of BrdU. We show that the concentration of BrdU used during cell culture has no effect on SCE rates and that SCE rates also do not increase when BrdU is present in DNA template strands. We also show that BS cells do display spontaneously elevated SCE rates that are not affected by the presence BrdU in cell culture medium or in DNA template strands. These results substantiate that SCEs play a biological role in cells and are not artefacts induced by the method used to detect them.

## MATERIALS AND METHODS

### Cell culture

The following cell lines were obtained from the Corriell Cell Repository: GM07492 (primary fibroblasts, normal), GM03402 (primary fibroblasts, BS), GM12891 (EBV-transformed lymphoblasts, normal) and GM16375 (EBV-transformed lymphoblasts, BS). Fibroblasts were cultured in Dulbecco's modified Eagle's medium (Life Technologies) supplemented with 15% v/v fetal bovine serum (Sigma Aldrich) and 1% v/v penicillin-streptomycin (Life Technologies), lymphoblasts in RPMI1640 (Life Technologies) supplemented with 15% v/v FBS and 1% v/v penicillin-streptomycin. All cells were cultured at 37°C in 5% CO_2_. BrdU (Invitrogen) was added to cultures at indicated concentrations for indicated periods of time.

### Flow cytometry

Cells were harvested after the BrdU pulse, and nuclei were isolated by suspending cells in nuclei isolation buffer (100 mM Tris–HCl pH7.4, 150 mM NaCl, 1 mM CaCl_2_, 0.5 mM MgCl_2_, 0.1% NP-40 and 2% bovine serum albumin). Nuclei were stained with Hoechst 33258 (Life Technologies) and propidium iodide (Sigma Aldrich) at final concentrations of 10 μg/ml. Nuclei were analyzed and sorted based on low PI (G1 phase) and low Hoechst (BrdU-induced quenching, see also Figure 2E) fluorescence on a MoFlo Astrios cell sorter (Beckman Coulter) or a FACSJazz cell sorter (BD Biosciences) directly into 5 μl Pro-Freeze-CDM NAO freeze medium (Lonza) + 7.5% DMSO, in 96-well skirted polymerase chain reaction (PCR) plates (4Titude). Sorted nuclei were stored at −80°C.

### Library construction

Library preparation was performed using the protocols previously described (21) with the following modifications. Enzymatic reactions were performed in smaller volumes but with the same enzyme and buffer concentrations. All clean-ups were performed using AMPure XP magnetic beads (Agencourt AMPure, Beckman Coulter). After adapter ligation and PCR, clean-ups with magnetic beads were performed twice using a 1.2 volume of beads. All pipetting was performed using the Bravo Automated Liquid Handling Platform (Agilent).

### Illumina sequencing

Libraries were pooled for sequencing and 250- to 450-bp size range fragments were purified using a 2% E-Gel Agarose Gel (Invitrogen). DNA quality was assessed and quantified on a High Sensitivity dsDNA kit (Agilent) on the Agilent 2100 Bio-Analyzer and on the Qubit 2.0 Fluorometer (Life Technologies). For sequencing, clusters were generated on the CBot (HiSeq2500) and single-end 50 bp reads were generated were generated using the HiSeq2500 sequencing platform (Illumina).

### Bioinformatic analysis

Indexed bam files were aligned using bowtie2 (22) and further analyzed using the BAIT software package (23). SCEs were detected by BAIT and their presence was confirmed manually.

## RESULTS

### Bloom Syndrome cells display high SCE rates during first division with BrdU

In order to assess any potential effect of BrdU on SCE rates, we first established baseline SCE rates in both normal and BS cells. We used primary fibroblasts and EBV-transformed lymphoblasts from healthy donors and BS patients for our analysis. Logarithmically growing cells were pulsed with 40 μM for one cell division (18 h for fibroblasts, 24 h for lymphoblasts). Cells were harvested directly after the BrdU pulse, nuclei suspensions were made and single nuclei were sorted by means of flow cytometry. Nuclei from cells in G1 phase (low PI fluorescence) that had incorporated BrdU into their DNA (low Hoechst fluorescence) were sorted and Strand-seq libraries were made. SCEs were detected by the BAIT analysis software (23) and confirmed by visual inspection (see also Supplementary Figure S1).

**Figure 1.**
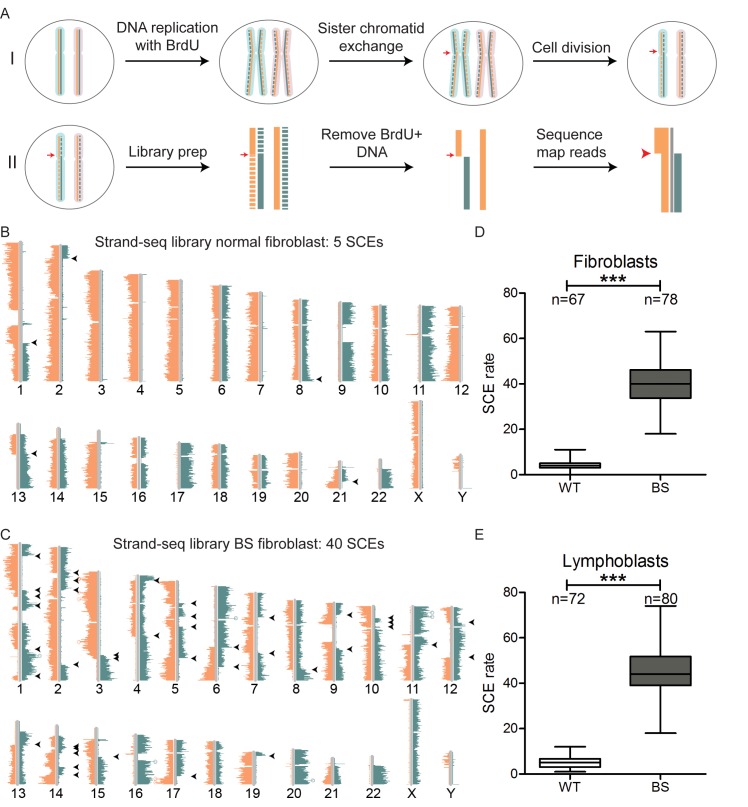
Strand-seq confirms high SCE rates in Bloom Syndrome (BS) cells. (**A**) Schematic diagram explaining the principle of Strand-seq. (I) A single cell with a single chromosome is represented. Homologs are shown in blue and pink shading. For each chromosome, the Watson (negative) strand is indicated in orange and the Crick (positive) strand in green. Cells are pulsed with BrdU for one cell cycle, causing newly synthesized strands during DNA replication to become labeled with BrdU (dashed lines), while the template strands remain unlabeled (solid lines). An SCE on one homolog (red arrow) will cause template strands to be exchanged between sister chromatids, leading each to have partial Watson and Crick template strands after cell division. (II) Single cells are sorted, DNA is isolated and a sequencing library is constructed. During library construction, all BrdU-labeled DNA is removed by treatment with Hoechst + UV radiation. DNA template strand fragments are amplified and sequenced and reads are mapped to the chromosome based on orientation. An SCE is detected as a switch from Watson to Crick, or *vice versa*, on one of the template strands (red arrow). (**B** and **C**) Representative Strand-seq ideograms made from a normal (B) and a BS fibroblast (C). Orange and blue lines correspond to reads aligning to the Watson and Crick strands, respectively. Chromosome numbers are indicated under each ideogram. SCEs are indicated by black arrowheads. (**D** and **E**) Average SCE rates for normal and BS fibroblasts (D, *P* = 5.3*10^−47^) and lymphoblasts (E, *P* = 2.8*10^−51^). *P*-values were calculated using t-test.

Two representative libraries made from a normal fibroblast (Figure 1A) and a BS fibroblast (Figure 1B) are depicted, displaying 5 and 40 SCEs, respectively. Average SCE rates were calculated across 67–80 Strand-seq libraries made from normal and BS fibroblasts (Figure 1C) and normal and BS lymphoblasts (Figure 1D). These results show that although there are minor differences between the absolute SCE rates detected in the fibroblasts and lymphoblasts, both BS cell lines display roughly a tenfold increase in SCE rates compared to their normal counterparts during the first cell division with BrdU. These results are in agreement with previously published studies, which also showed ∼10-fold increase in the rate of SCE in BS cells. Interestingly, the average SCE rates detected here correspond to roughly half the baseline SCE rates found after two cell divisions in similar lymphoblast (17,18) and fibroblast (20) cells. This suggests that SCE rates are stable over two subsequent cell divisions, as previously suggested (19).

### BrdU concentration does not affect SCE rates in normal or Bloom Syndrome cells

**Figure 2.**
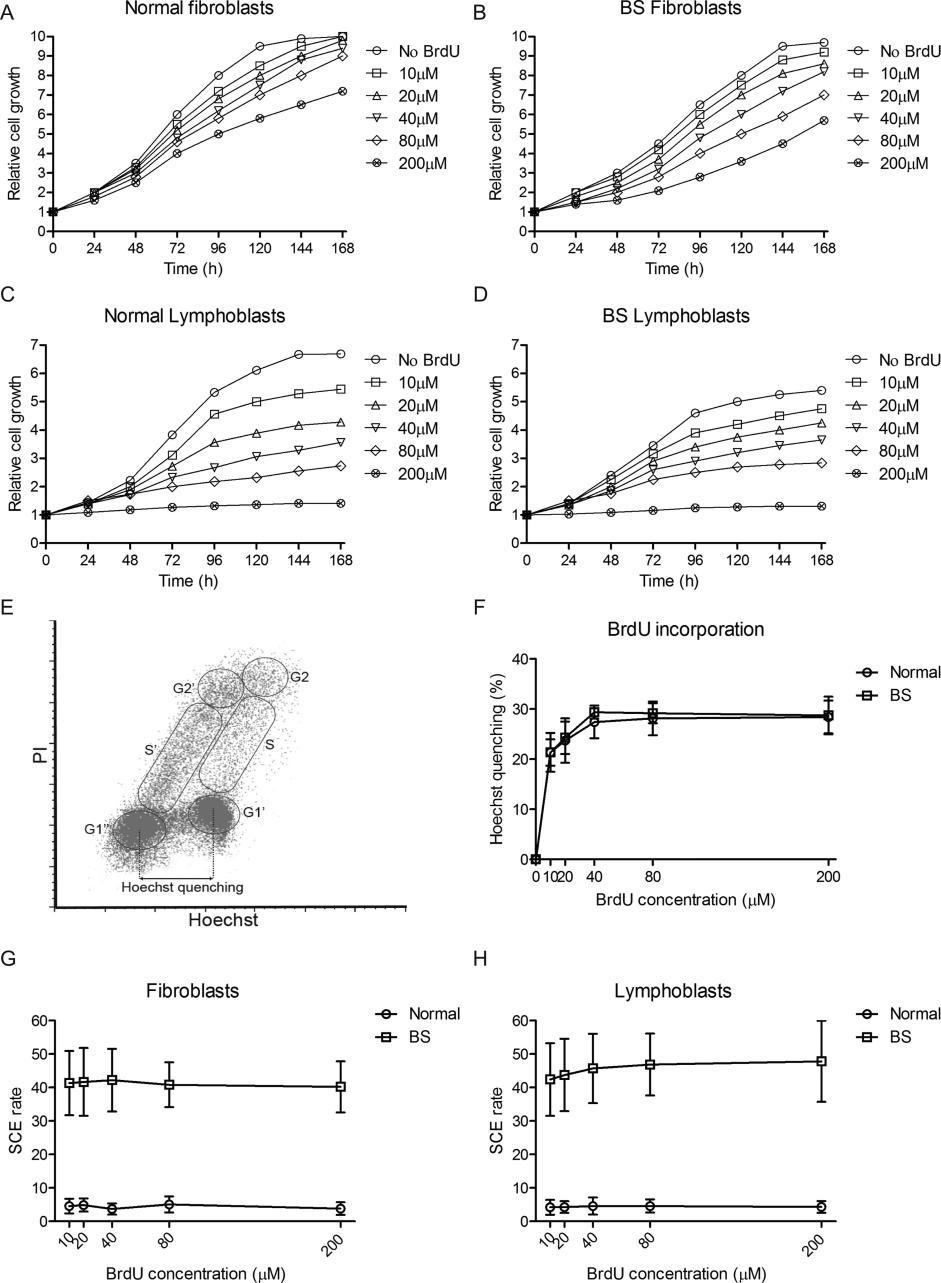
BrdU does not affect SCE rates in normal or BS cells. (**A**–**D**) Relative growth curves for normal fibroblasts (A), BS fibroblasts (B), normal lymphoblasts (C) and BS lymphoblasts (D). Number of live cells for each time point was normalized to t0, each panel represents a single replicate experiment. (**E**) Example Hoechst and PI fluorescence of asynchronously growing BrdU-labeled, measured by flow cytometry. Cell cycle stages can be distinguished based on PI staining intensity, BrdU-labeled (G1’, S’, G2’) and unlabeled nuclei (G1, S, G2) based on Hoechst intensity. Relative Hoechst quenching is calculated at the decrease in fluorescence between G1 nuclei with (G1’) and without (G1) BrdU labeling. (**F**) Relative Hoechst quenching of nuclei from normal and BS lymphoblasts pulsed with different concentrations of BrdU. (**G**) Average SCE rates across Strand-seq libraries made from normal and BS fibroblasts pulsed with different concentrations of BrdU (Normal: *n* = 15–24, BS: *n* = 16–28). (**H**) Average SCE rates across Strand-seq libraries made from normal and BS lymphoblasts pulsed with different concentrations of BrdU (Normal: *n* = 10–22, BS: *n* = 18–25).

Next, we investigated whether incorporation of BrdU into newly formed DNA strands could induce SCEs. Both normal and BS cells were cultured with increasing concentrations of BrdU. First, the effect of BrdU on proliferation was determined by culturing cells in increasing concentrations of BrdU and tracking proliferation over the course of 7 days. Fibroblasts were seeded at 10% confluency, lymphoblasts at 200 000 cells/ml. The number of live cells was counted using the trypan blue exclusion method every 24 h or until cultures reached confluency. The results (Figure 2A–D) show that BrdU caused a concentration-dependent decrease of cell proliferation in all cell lines tested. Quite strikingly, the effect of BrdU is much stronger in lymphoblasts than in the fibroblast lines; both lymphoblast lines show hardly any proliferation at 200 μM, while both fibroblast lines still continue to divide at this dose. Finally, both BS cell lines displayed decreased cell growth compared to normal cells, independent of BrdU. In addition, BS cells do not appear to be hypersensitive to the presence of BrdU.

BrdU is thought to affect SCE frequencies when it is present in the DNA, so therefore we attempted to confirm that higher doses of BrdU also lead to higher BrdU incorporation into the DNA during cell division. Because fluorescence of Hoechst bound to DNA decreases when BrdU is present in the DNA (24), we decided to measure Hoechst fluorescence by means of flow cytometry and use relative Hoechst quenching as an indicator of the level of BrdU incorporation into the DNA. Figure 2E shows an example of Hoechst and PI staining of asynchronous nuclei labeled pulsed with BrdU. Relative Hoechst quenching, calculated at the decrease in Hoechst fluorescence between BrdU-labeled and unlabeled cells, is a measure for the amount of BrdU incorporation into the DNA. In order to determine the effect of BrdU concentration, normal and BS lymphoblasts were pulsed with the same range of BrdU concentrations as above for 24 h, after which cells were harvested, nuclei were isolated and stained with PI and Hoechst and fluorescence was measured by flow cytometry. Hoechst quenching was calculated and the results (Figure 2F) show that there is a dose-dependent effect of BrdU concentration on Hoechst quenching in both normal and BS cells.

In order to determine if BrdU does indeed induce SCEs, all four cell lines were pulsed with 10–200 μM BrdU for one cell division and Strand-seq was performed to assess SCE rates under the different conditions. The results confirmed the high SCE rates in BS cells at each BrdU concentration, but we did not detect any significant effect of BrdU concentration on SCE rates in any of the cell lines (Figure 2G and H).

### Presence of BrdU in DNA template strands does not induce SCEs in normal or BS cells

**Figure 3.**
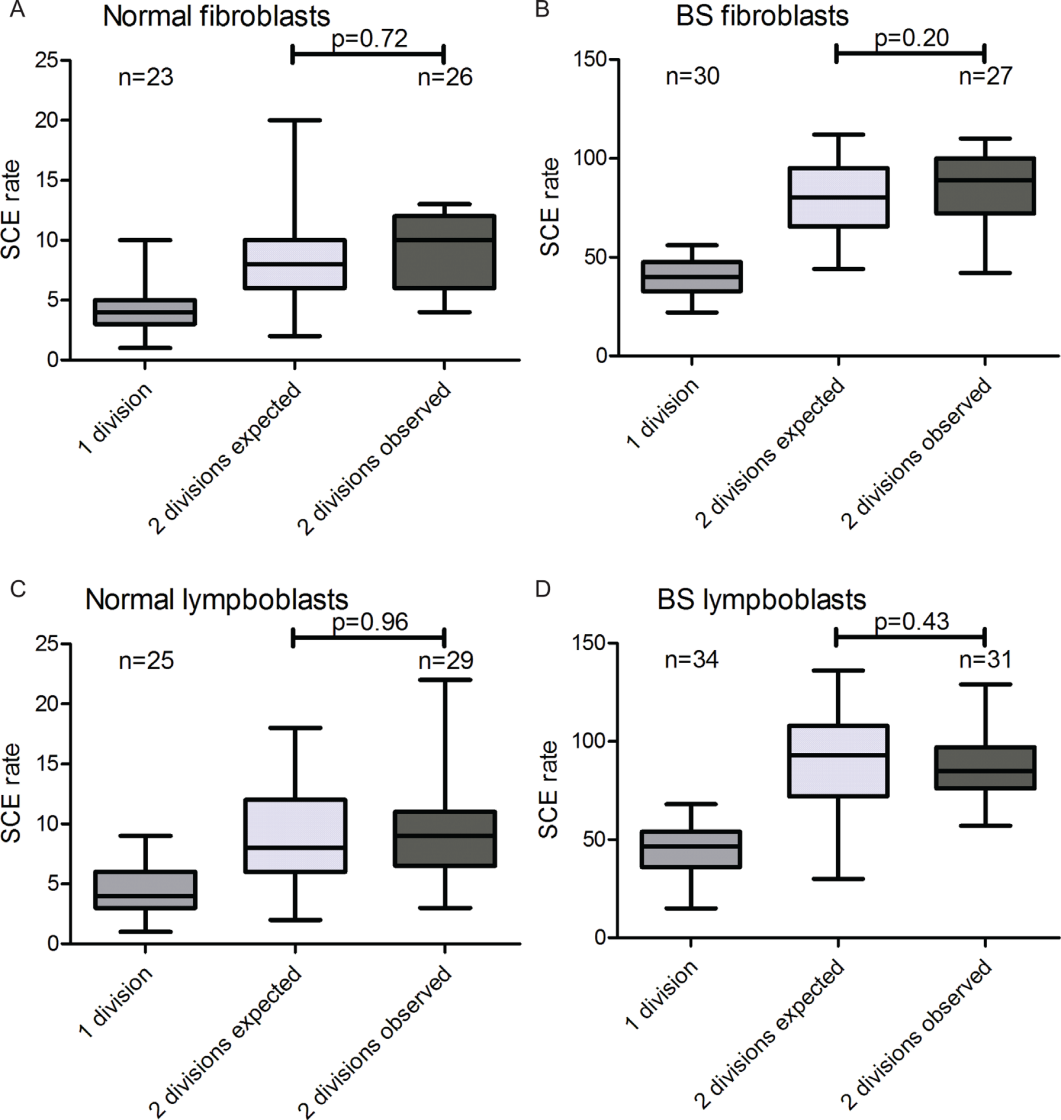
No increase in SCE rates during second cell division. (**A**–**D**) Average SCE rates after one and two cell divisions in BrdU as expected and observed for (A) normal fibroblasts, (B) BS fibroblasts, (C) normal lymphoblasts (D) and BS lymphoblasts. *P*-values were calculated using *t*-test.

It has previously been proposed that DNA replication over BrdU-substituted DNA induces SCEs (10). Although we show that the presence of BrdU in cell culture medium does not affect SCE rates, we cannot exclude any effect of BrdU in DNA template strands based on these results. Fortunately, Strand-seq can also be used to detect SCEs after two cell divisions in BrdU. However, it is not possible to distinguish between SCEs that occurred during the first or the second cell cycle. In order to properly assess SCE rates during the second cell cycle, fibroblasts and lymphoblasts were pulsed with 40 μM BrdU for 36 or 48 h respectively, and single nuclei that underwent either 1 or 2 cell divisions were sorted. Strand-seq was performed on these nuclei after which first and second division libraries could be distinguished based on strand inheritance patterns. SCE rates in first division libraries were doubled to simulate a second cell division without induction of extra SCEs, and these expected SCE rates were compared to observed SCE rates in second division libraries (Figure 3A–D). The observed SCE rates matched the expected SCE rates in each of the four cell lines, supporting the notion that BrdU in DNA template does not induce SCEs in either normal or BS cells at concentrations typically used for SCE detection.

## DISCUSSION

The role BrdU plays in SCE formation has been debated for several decades, but has not been resolved convincingly. Here we use Strand-seq to show that BrdU does not induce SCEs in either normal or BS cells and that Strand-seq can be used to detect spontaneously occurring SCEs in cultured cells.

SCE detection by means of staining of metaphase spreads requires two rounds of DNA replication in the presence of BrdU. Because of this, the number of SCEs that occurred during a first cell cycle had to be estimated based on complex staining patterns in second or third cell cycle metaphase spreads. Using Strand-seq, it is possible, for the first time, to directly detect SCEs after one cell division. Our results indicate firstly that SCE frequencies are elevated in both BS fibroblasts and lymphoblasts compared to their normal counterparts. Secondly, the concentration of BrdU used during cell culture has no effect on SCE frequencies. Thirdly, SCE frequencies do not increase during a second cell cycle in the presence of BrdU in either normal or BS cells. Based on these results, we conclude that BS cells display spontaneously elevated SCE rates and that BrdU has no effect on SCE rates in either normal or BS cells.

These results disagree with the generally accepted notion that BrdU does induce SCEs, either during incorporation into nascent DNA strands or during DNA replication over BrdU-substituted DNA (7–11). Although cytogenetic SCE detection is possible at lower BrdU concentrations than those required for Strand-seq, several studies did report an effect of BrdU at similar concentrations to the ones used here (8,9,19). What factors could explain these different results? It has been shown that the presence of BrdU in DNA increases sensitivity of cells to a wide range of chemicals, including mitomycin C (2), Hoechst (7) and ethylnitrosourea (25), as well as UV radiation (26) and DNase I (27). This suggests that BrdU itself does not induce SCEs in cells, but is capable of sensitizing cells to agents that do. The power of Strand-seq allows us to detect SCE rates after one cell division in BrdU, thus minimizing any outside effect on SCE frequencies. This also highlights the importance of minimizing exposure to exogenous sources of DNA damage while culturing cells in BrdU.

It has previously been suggested that BS cells only display elevated SCE rates when replicating DNA over BrdU-labeled template strands, suggesting that the high SCE rates are induced by BrdU and do not occur spontaneously (17,18). In other studies, no such effect was detected and it was concluded that BS cells do show spontaneously elevated SCE rates (19,20). It is unclear exactly why different results were obtained in these studies, but they were obtained based on highly complex staining patterns observed in metaphase spreads after two or three rounds of DNA replication in BrdU. This method is susceptible to misinterpretation of results when SCEs are not properly assigned to the cell cycle during which they occurred (28). Misidentification is even more likely to occur in BS cells due to the high SCE rates and multiple SCEs occurring in close proximity, leading to metaphase spread staining patterns that cannot be reliably analyzed.

One major difference between these previously reported studies are the cell types used for experiments: Epstein-Barr virus (EBV) transformed B lymphoblastoid cell lines (17,18), primary lymphocytes (19) and primary fibroblasts (20). It has been suggested that the different results reflect an effect of cell transformation by EBV (19,20). However, we used both EBV transformed lymphoblasts and primary fibroblasts in this study and we found no differences in relative SCE rates and effect of BrdU on SCE rates. The only differences we observed are that both lymphoblast cell lines displayed slightly higher SCE rates than the fibroblast cell lines and that BrdU had a larger effect on cell proliferation in the lymphoblasts. This phenotype was seen in both the normal and BS lymphoblasts, suggesting it was caused by an intrinsic difference, possibly reflecting the ongoing oncogenic stress that occurs as the result of EBV-transformed nature of the cells.

Based on these results, we conclude that BS cells display spontaneously elevated SCE rates and that this reflects high levels of genomic instability in patient cells that likely contributes to the wide range of symptoms associated with BS, including the strong cancer predisposition seen in patients (29). Finally, we show that Strand-seq can be used to detect spontaneously occurring SCEs at high resolution, making it a powerful tool for studying genomic instability at the single cell level.

## ACCESSION NUMBER

Sequencing data have been deposited in the European Nucleotide Archive under accession number PRJEB13795.

## Supplementary Material

SUPPLEMENTARY DATA
