# Supplementary material for: Bromodeoxyuridine does not contribute to sister chromatid exchange events in normal or Bloom syndrome cells
Source: Nucleic Acids Res. 2016 May 16;44(14):6787–93. doi: 10.1093/nar/gkw422 (PMC5001594; doi:10.1093/nar/gkw422)
Supplement: SUPPLEMENTARY DATA [file supp_44_14_6787__index.html]

Bromodeoxyuridine does not contribute to sister chromatid exchange events in normal or Bloom syndrome cells — SUPPLEMENTARY DATA 

# Bromodeoxyuridine does not contribute to sister chromatid exchange events in normal or Bloom syndrome cells

## SUPPLEMENTARY DATA

- SUPPLEMENTARY DATA
