# Supplementary material for: Bromodeoxyuridine does not contribute to sister chromatid exchange events in normal or Bloom syndrome cells
Source: Nucleic Acids Res. 2016 May 16;44(14):6787–93. doi: 10.1093/nar/gkw422 (PMC5001594; doi:10.1093/nar/gkw422)
Supplement: SUPPLEMENTARY DATA [file supp_gkw422_nar-00917-x-2016-File005.pdf]

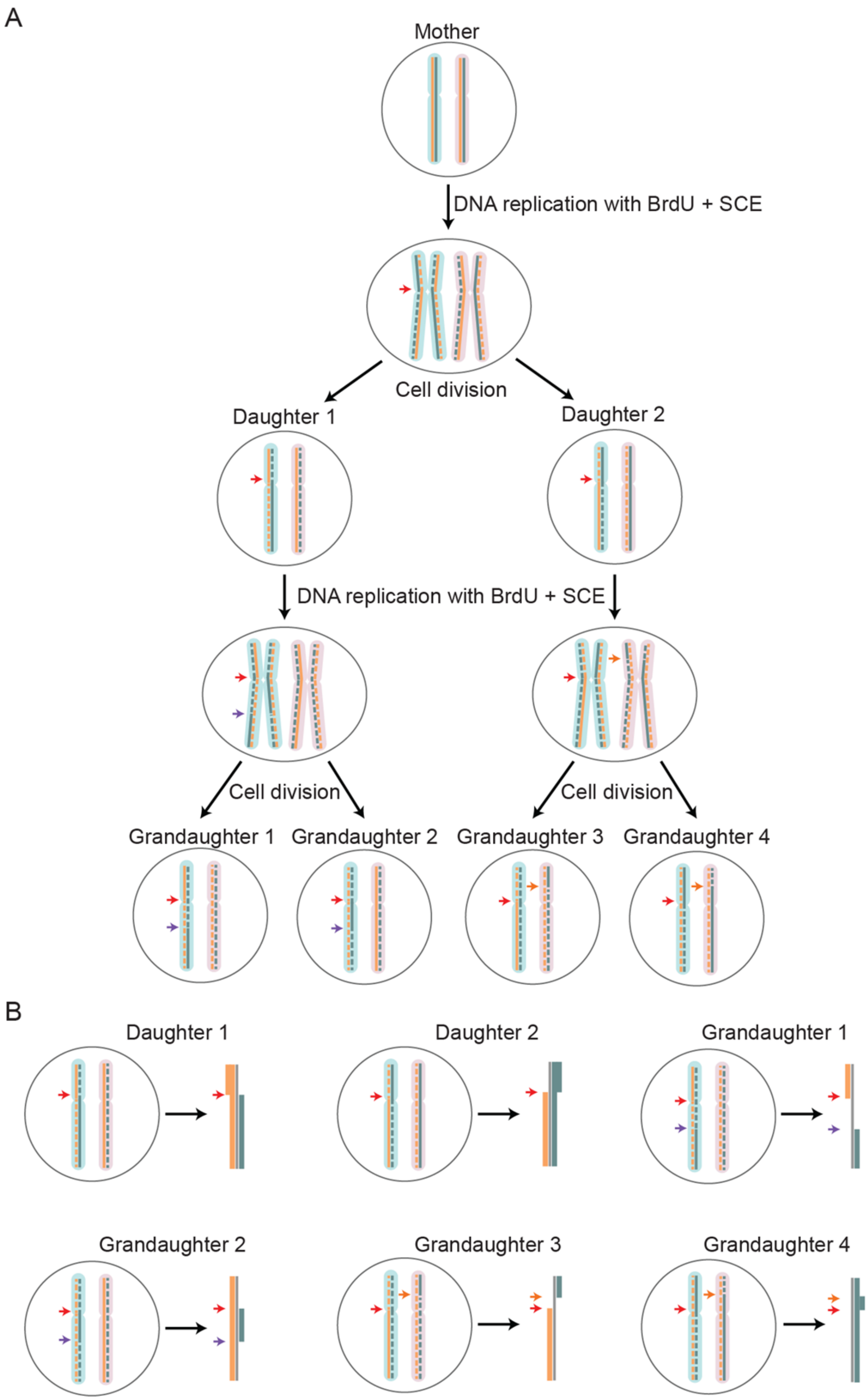
A
Mother
DNA replication with BrdU + SCE
Cell division
Daughter 1
Daughter 2
DNA replication with BrdU + SCE
Cell division
Cell division
Grandaughter 1
Grandaughter 2
Grandaughter 3
Grandaughter 4
B
Daughter 1
Daughter 2
Grandaughter 1
Grandaughter 2
Grandaughter 3
Grandaughter 4

Figure S1. SCE detection after one and two cell divisions using Strand-seq. (A) For Strand-seq, cells are typically pulsed with BrdU for one cell division, generating BrdU-labelled strands during DNA replication. A sister chromatid exchange (red arrow) in the paternal homolog (indicated in blue) disrupts the template strand, yielding two partially substituted DNA strand per sister chromatid, while the unaffected maternal homolog (indicated in pink) has one BrdU-labelled and one unlabelled strand per sister chromatid. A second cell division with BrdU normally yields chromosomes with either one or two BrdU-labelled DNA strands, while SCEs I either the first or second (purple and orange arrows) cell division lead to partially BrdU-labelled strands. (B) Strand-seq profiles for both daughter cells and the four graunddaughter cells. After one cell division (daughter cells), SCEs are detected as switches in DNA template strand state from Watson to Crick (daughter 1) or Crick to Watson (daughter 2). By contrast, SCEs in second division libraries (granddaughter cells) can be detected as gain or loss of information from either DNA template strand. In this case, first and second division SCEs cannot be distinguished from each other.
